# Flexural Properties and Fracture Behavior of Nanoporous Alumina film by Three-Point Bending Test

**DOI:** 10.3390/mi8070206

**Published:** 2017-06-27

**Authors:** Jung-Hsuan Chen, Wen-Shiang Luo

**Affiliations:** 1 Department of Industrial Education, National Taiwan Normal University, 162, Sec.1, Heping E. Rd., Taipei 106, Taiwan; 2 Passive BU Department, Cyntec Company, 2, Yanfa 2nd Rd., Hsinchu 300, Taiwan; WenShiang.luo@cyntec.com

**Keywords:** flexural property, fracture, nanoporous, alumina film, plastic deformation

## Abstract

This study investigated the influence of porosity on the flexural property of a nanoporous alumina film. When the porosity of the alumina film increased, both bending strength and modulus declined. The results from the bending test revealed that the setting of the film during the bending test had significant influence on the flexural property. Fracture only occurred when the porous side of the alumina film suffered tensile stress. The ability to resist fracture in the barrier layer was higher than in the porous side; the magnitude of the bending strength was amplified when the barrier layer sustained tensile stress. When the porous layer suffered a tensile stress, the bending strength decreased from 182.4 MPa to 47.7 Mpa as the porosity increased from 22.7% to 51.7%; meanwhile, the modulus reduced from 82.7 GPa to 17.9 GPa. In this study, the most important finding from fractographic analysis suggested that there were a localized plastic deformations and layered ruptures at the porous side of the alumina film when a load was applied. The fracture behavior of the nanoporous alumina film observed in the present work was notably different from general ceramic materials and might be related to its asymmetric nanostructure.

## 1. Introduction

The research and development of nanoporous templates has seen an increase in the number of participating industries and academic organizations. Anodic aluminium oxide (AAO) is among the most extensively used. Research on the formation procedure and structure analysis on the porous alumina film has been studied for more than 50 years [1,2,3,4,5]. The structure of the film includes two parts: a porous layer and barrier layer. The porous layer is a hexagonal honeycomb cell in a regular arrangement, with cylinder-like pores in the nanometer scale. The barrier layer is dense and forms at the boundary of the aluminium substrate and alumina film [6]. Extensive application of this porous alumina film exists because of its nanometer dimension and special structure. It has been widely used as a template for the preparation of metal and ceramic nanowires [7,8,9,10], fuel cells [11], and porous Si or TiO_2_ structures [12,13]. In recent years, the porous alumina film has drawn a great deal of attention to the fabrication of nanoelectronic devices, such as gas sensors [14,15] and field emission devices [16]. A suitable mechanical property is a prime request for producing reliable electronic devices. Therefore, it will be important to know the mechanical properties and fracture behavior of the porous alumina film.

Alumina is a ceramic material that suffers from the lack of elongation, weakness, and brittleness. The Young’s modulus of bulk alumina is approximately 350 to 390 GPa [17]. According to nanomaterial literature, when the materials’ dimensions shrink to nanoscale, they are expected to exhibit very different properties from their bulk forms. Moreover, the AAO film has a nanoporous and asymmetric structure, which will be an attractive aspect of how this changes the mechanical properties of the nanoporous alumina film. Most studies on the mechanical properties of AAO films were focused on using the measurement technique of nano-indentation, which was a popular tool for nanotechnology [18,19,20,21]. Alcala et al. [18] were the first group that performed research on AAO films with Al substrates by use of the nanoindenter. These researchers observed that the hardness of the AAO film was 7 GPa, which was lower than that of sapphire (26 GPa). The Young’s modulus of the AAO film was 122 GPa, which was about one-third of the value for general crystalline alumina. Xia et al. [22] measured mechanical properties of AAO films without Al substrates by use of the nanoindenter. The hardness and Young’s modulus were 5.2 GPa and 140 GPa, respectively, and similar to Alcala’s results. Furthermore, Xia and his coworkers also reported that the existence of moisture would affect the hardness but Young’s modulus and the deformation of the AAO film occurred after the experiment of nanoindentation. How hardness changes for AAO films with various pore sizes, porosities, and crystal structures were offered by Gall et al. [23]. They noted that the hardness and Young’s modulus of crystalline AAO templates were higher than amorphous ones. 

Regarding bending properties of AAO films, an atomic force microscope (AFM) or a nano-universal testing machine (UTM) were usually used in the bending test. In Jeon’s study [24], bending tests were carried out on cantilever beams by pressing AFM tips, and the results were compared with three-point bending tests conducted by using the nano-UTM. They found that Young’s modulus was the same, approximately 30 to 40 GPa, in both measurements and the existence of clogged and open layers did not affect the stress distribution in the porous layer or its modulus. Choi et al. [25] observed that the bending moduli of the nanohoneycomb structures decreased nonlinearly as a function of porosity from the results of bending tests in AFM.

Although many works have been completed to date, few studies measuring the flexural properties of AAO films, especially fractography studies, have been reported. Therefore, more research needs to be conducted to understand the flexural properties of AAO films more thoroughly. The present study was carried out to determine the relationship between the porosity and the flexural property of AAO films. To establish a reasonable inference to examine the flexural property, the configuration of the bending test and fracture behavior of the AAO film were also discussed. The results of this study could be useful in understanding the fracture mechanism of the nanoporous alumina film.

## 2. Experimental Method

In this study, AAO films were produced by a two-step anodization method in an oxalic acid solution. General commercial aluminium foil with a purity of 99.7 wt % was used to produce AAO films. The aluminium sheets were first annealed at 673 K for 3 h to release the mechanical stresses and then electropolished. After electropolishing, the two-step anodization process followed. In the first anodization process, the treated aluminium sheet was anodized in 0.3 M oxalic acid solution under a voltage of 40 V at 20 °C for one hour and a platinum plate was used as the cathodic electrode. The pre-formed alumina was removed by wet etching in a mixture of 6 wt % phosphoric acid and 2 wt % chromic acid at 60 °C until the alumina entirely dissolved. The sample was anodized again under the same condition as the first anodization but for 7 h. Hexagonally ordered pores were obtained on the aluminium surface after the entire anodization process. The subsequent processes were removing the aluminium substrate and pore widening. The purpose of removing the aluminium substrate was to separate the AAO films and the substrate. The remaining aluminium was removed in a saturated CuCl_2_ solution based on the following reaction: 2Al + 3Cu^2+^→ 2Al^3+^ + 3Cu. Finally, the sample was thoroughly cleaned in distilled water. Pore widening of the AAO film using chemical etching was investigated in this work. The process of the pore widening was to enlarge the pores of the AAO films under fixing the thickness. The pore sizes and porosities were controlled by choosing suitable etching times. The etching solution was a 5 wt % phosphoric acid solution and the AAO films were immersed in the solution at 25 °C for 20 to 60 min. 

Mechanical properties of the AAO films were measured by a three-point bending test, which was a common measurement test for ceramic materials. The dimensions of the AAO film were 30 mm in length and 9 mm in width. The thickness of the AAO film was approximately 57 μm. The interval between two supporters in the three-point bending test was 20 mm and the loading span was 10 mm. The spans and three-point fixture configuration followed the designation described by the ASTM standard test method C1161-13 [26]. The crosshead speed of the three-point bending test was controlled at 0.01 mm/s and performed by an MTS Tytron 250 (MTS, Eden Prairie, MN, USA). 

In this study, surface morphologies of the AAO films and fractography analysis of the specimens after the three-point bending test were carried out by field emission scanning electron microscopy (FESEM JSM-6500F and 6700F, JEOL, Akishima, Tokyo, Japan). The surface images of the AAO films from SEM were used to calculate the porosities of the AAO film by the commercial image analysis software, Image-Pro Plus 6.0 (Media Cybernetics, Rockville, MD, USA).

## 3. Results and Discussion

**Figure 1:**
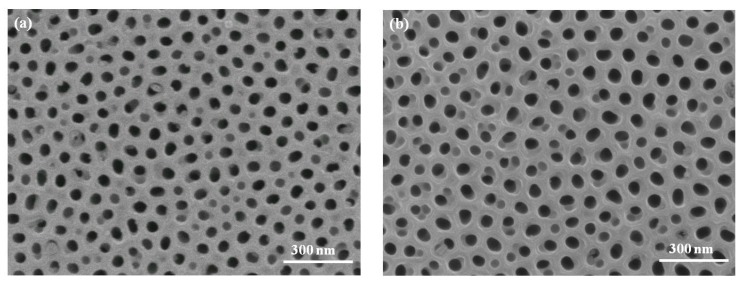

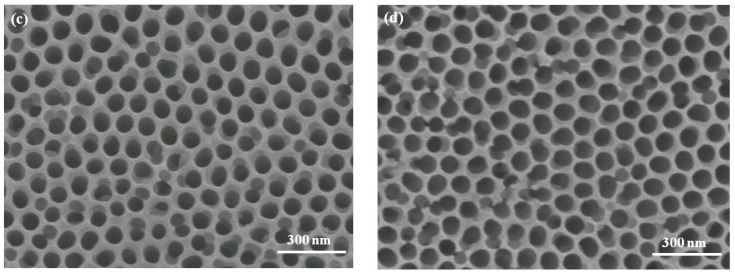
SEM images of the AAO films after the pore widening process: (**a**) N1 (no pore widening); (**b**) N2 (pore widening 20 min); (**c**) N3 (pore widening 40 min); (**d**) N4 (pore widening 60 min).

In this study, AAO films were produced by a two-step anodization method in an oxalic acid solution. The effect of porosity on the mechanical properties of the AAO film was investigated. Four AAO specimens were prepared for the three-point bending test in this study. The porosity of each specimen was 22.7, 23.9, 32.6, and 51.7%. The detailed conditions of the specimens were presented in Table 1. All specimens from N1 to N4 were prepared under the same anodization condition with a thickness of approximately 57 μm, which was measured using SEM. To obtain an AAO specimen with different porosity, a pore widening process employing chemical etching was applied to specimens N2, N3, and N4. When the etching time of the AAO films, immersed in the phosphoric acid solution, increased, the porosity of the AAO film showed a tendency to increase. Figure 1 displayed the morphologies of the AAO specimens after the pore widening process. When the pore widening time was longer than 40 min, the cell walls between pores became thinner and broke slightly, as shown in Figure 1c. More serious damage at the cell walls was observed when the pore widening time reached 60 min. 

**Figure 2:**
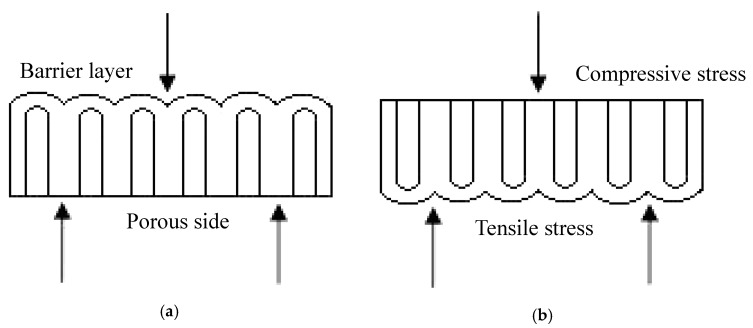
Two test types for the three-point bending test. (**a**) test type 1: porous layer suffered a tensile stress; and (**b**) test type 2: barrier layer suffered a tensile stress.

In the three-point bending test, the surface of the specimen, which was in contact with the loading pin, would be under a compressive stress and the other surface that touched the two supporting pins was under a tensile stress. In this study, two configurations of the AAO specimens were used in the three-point bending test. Figure 2a was test type 1, the porous layer of the AAO film suffered a tensile stress, while the barrier layer bore a compressive stress. In contrast, with test type 1, when the porous layer of the AAO film suffered a compressive stress, this configuration was test type 2, as given in Figure 2b.

**Figure 3:**
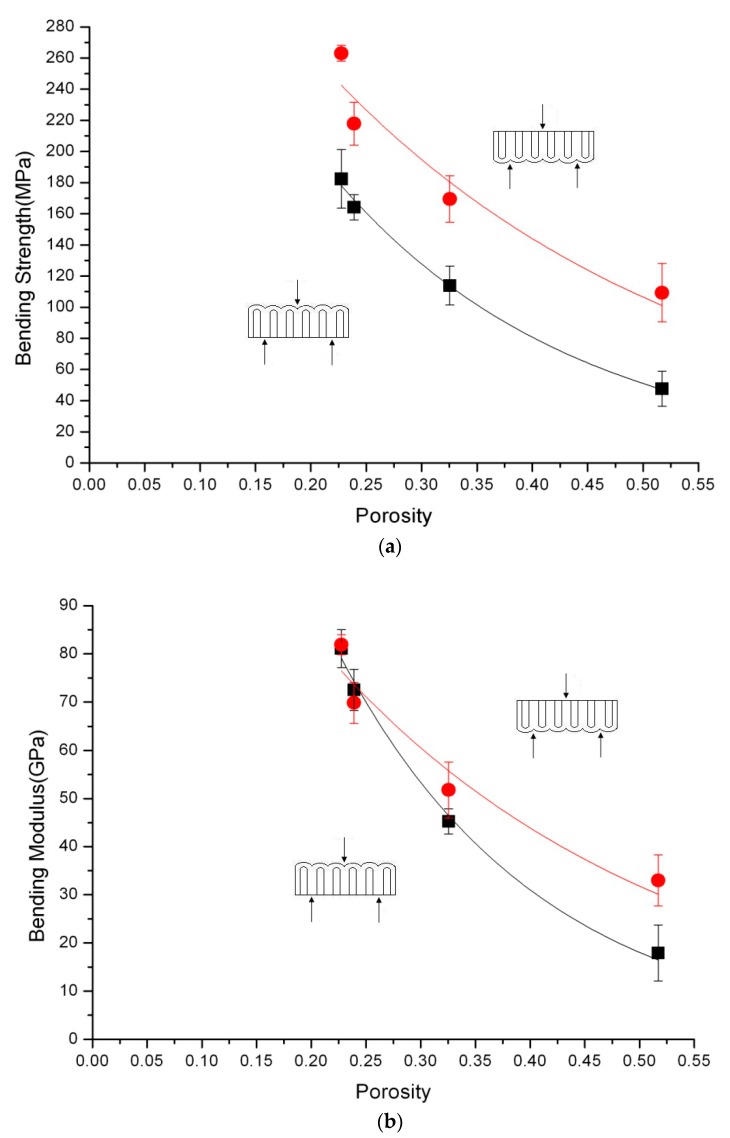
(**a**) Bending strength of AAO films calculated according to the Formula (1); (**b**) bending modulus of AAO films determined from the Formula (2).

Bending strength is calculated by a maximum force during the bending test using the formula

(1) $$\sigma =\frac{3FL}{2bh^{2}}$$

in which σ is the bending strength, *F* is the maximum force, *L* is the distance between the supporters, *b* is the width of the specimen, and *h* is the thickness of the specimen. Bending modulus is calculated by the slope of the elastic deformation region in the force-deflection diagram as

(2) $$E=\frac{kL^{3}}{4bh^{3}}$$

where *E* is the bending modulus and *k* is the slope in the force-deflection diagram. Both of these calculations obey the beam theory. The results of the three-point bending test are shown in Table 2 and Figure 3. 

No fracture occurred when test type 2 was performed. During the bending test, the deflection of the specimen exceeded 5 mm, which was considerably more than the thickness of the AAO film. Meanwhile, the shift of the specimen at the two supporters was observed. The cause of the shift was due to the large deflection of the specimen. When the applied force was removed, the AAO specimen could recover rapidly from the deflection. This indicated that the deformation of the specimen was still within the elastic range. Table 2 also showed that AAO films broke easily when test type 1 was adopted. The AAO film would fracture because the porous layer of the AAO film could not sustain the excessive tensile stress; consequently, the fracture began at the porous side and was located in the middle of the specimen. The result revealed that the barrier layer of the AAO film had the better resistance to tensile stress than the porous side of the film. For general ceramic materials, the capability of resisting tensile stress and compressive stress were different, and the compression resistance would be higher than the tensile one. In the three-point bending test, the fracture decision plane of general ceramic materials was the plane under the tensile stress. According to the results above, it could be seen that the porous layer of the AAO film under a tensile stress was the main factor for fracture during the three-point bending test. Furthermore, considering the asymmetric structure of the AAO film, the barrier layer of the AAO film was a dense alumina and would resist the larger tensile stress than the porous layer of the AAO film. That was the reason why all films using test type 2 would not be broken. Additionally, variations in porosities of the AAO films would not lead to fracture, even if the cell walls were thinner and damaged when the porous layer suffered the compressive stress. However, the thinner and broken cell walls weakened the mechanical properties of the AAO film. The bending stress of the AAO film decreased with the increase of the porosity in both test types, as shown in Figure 3a. The result appeared to be consistent with the characteristics of common ceramic materials [27]. 

Bending modulus was calculated by the slope of the elastic deformation region in the force-deflection diagram using Formula (2). The results reflected that the bending modulus of the AAO films, with the porosity less than 25%, had almost no difference between both test types. The results greatly resembled the research proposed by Gall et al. [23] and Jeon et al. [24]. Jeon and his coworkers described that there was no significant difference in the bending modulus between two three-point bending configurations of a nano-universal testing machine.

When the porosity of the AAO film was higher than 25%, different behaviors from those in previous studies could be observed in our work. The bending modulus of test type 1, where the porous layer was suffering tensile stress markedly decreased compared to test type 2. It was suspected that there were increasing numbers of defects in the porous layer of the AAO film as the time of pore widening increased. These defects might be the crack tips during the bending test and caused the stress concentration, leading to declines of the bending strength and bending modulus.

**Figure 4:**
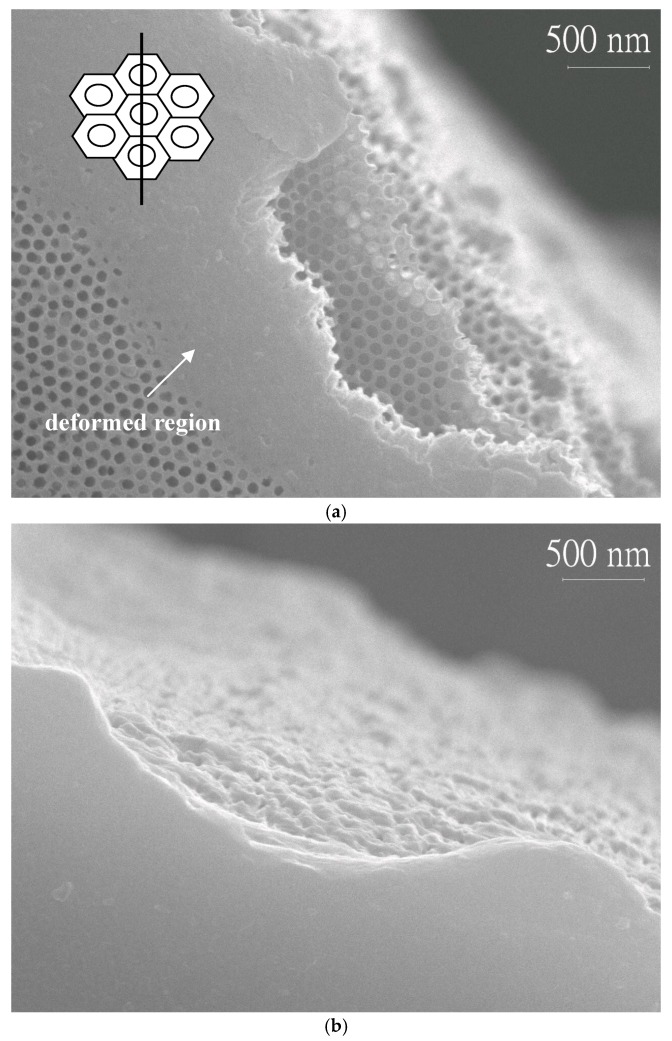
Fracture morphologies of the specimen N3 using the test type 1, which the porous layer suffered the tensile stress: (**a**) SEM image of the porous layer; and (**b**) SEM image of the barrier layer. The inset in the image (**a**) showed the pore configuration and fracture position.

**Figure 5:**
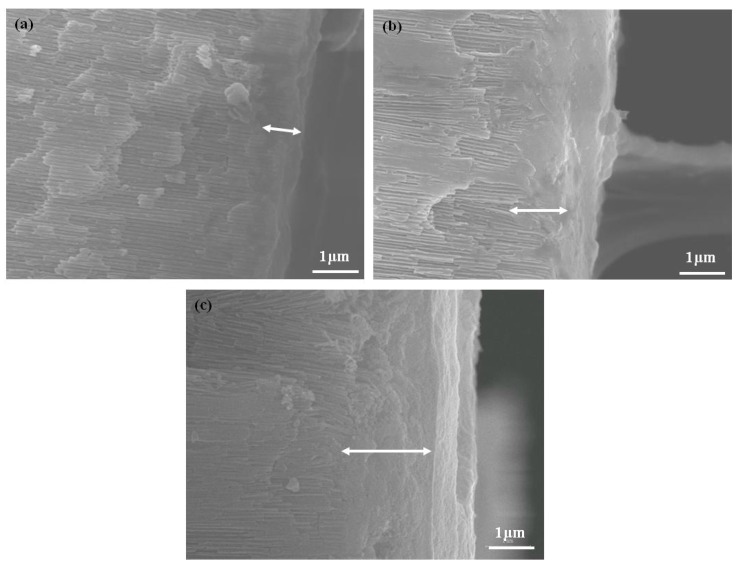
Cross-section images of fractured specimens showed that plastic deformations could be found in AAO films undergoing the three-point bending test as test type 1. SEM images at the porous sides of the fractured specimens (**a**) N2; (**b**) N3; and (**c**) N4, displayed deformed regions which were marked by left right arrows.

Fractography is usually used to determine the root cause of failure or examine the crack growth behavior by studying the characteristics of a fractured surface. In this study, fractographic inspection was performed by an SEM at a finer scale and a higher resolution. Figure 4 provided the fractured surfaces from the porous layer and the barrier layer of the AAO specimen N3 after a three-point bending test, with the porous layer being under a tensile stress. Plastic deformations along the edge of the fracture were observed in both surfaces, as well as layered ruptures. These phenomena were not typical features of fracture in most ceramics. It was believed that the AAO film could no longer withstand the maximum stress, being deformed at the region touching the loading pin, and broke finally. The deformation range noted in Figure 4a was 1~2 μm at the edge of fracture and only occurred at the surface layer because the porous structure could be seen at the inner of the film. The inset in Figure 4a presented schematically that the cracks may begin at the cell wall initially and propagated across the pores, resulting in the eventual fracture. This model could also explain why the bending strength and modulus declined noticeably for specimens N3 and N4, due to the thinner and damaged cell walls. Cross-section images of the fractured specimens, N2, N3, and N4, were shown in Figure 5. At the porous side of the film, the deformed region, marked by the left right arrow, was larger than 1 μm and enlarged with the increase of pore widening times. Specimen N4 had the maximum plastic deformation range.

## 4. Conclusions

Flexural properties, such as bending strength and bending modulus of the nanoporous alumina film, were investigated in this study. AAO films were produced by a two-step anodization method in an oxalic acid solution. Porosities of the four AAO specimens were 22.7, 23.9, 32.6, and 51.7%, respectively, with the thickness in all specimens being approximately 57 μm. In terms of the experimental results, the porosity of the alumina film and the film setting for the three-point bending test had significant effects on the flexural properties. The porous layer of the AAO film was the fracture decision plane because the fracture only occurred when the porous layer of the film suffered a tensile stress (test type 1). The bending strength decreased from 182.4 MPa to 47.7 MPa when the porosity increased from 22.7% to 51.7%; meanwhile, the modulus reduced from 82.7 GPa to 17.9 GPa. Additionally, there was no obvious difference in the bending modulus measured using both settings of the AAO films (test types 1 and 2) when the porosity was lower than 25%. The decline rate of the bending modulus was faster in the specimen with the porous layer under a tensile stress when the porosity of the AAO film was higher than 25%. SEM inspections of the fractured specimens showed that plastic deformations and layered ruptures occurred for the duration of stress application proceeding fracture. These phenomena were not the typical features of fracture in most ceramics. It was believed that the fracture behaviors observed in this work were likely due to the contribution of the AAO’s nanostructure.

## Figures and Tables

**Table 1:** Porosities of AAO films with different pore widening times in the phosphoric acid solution.

Sample	Pore Widening Time	Porosity
N1	0 min	22.7 ± 0.4%
N2	20 min	23.9 ± 0.7%
N3	40 min	32.6 ± 1.1%
N4	60 min	51.7 ± 2.3%

**Table 2:** Experimental results of three-point banding test.

Test Type	Sample	Bending Stress (Mpa)	Bending Modulus (Gpa)	Fracture
Type 1	N1	182.38 ± 18.80	82.70 ± 2.92	Yes
	N2	164.17 ± 8.09	72.51 ± 4.29	Yes
	N3	113.87 ± 12.54	45.26 ± 2.59	Yes
	N4	47.67 ± 11.12	17.92 ± 5.81	Yes
Type 2	N1	263.08 ± 5.06	81.09 ± 2.09	No
	N2	217.87 ± 13.88	69.84 ± 4.24	No
	N3	169.49 ± 14.94	51.78 ± 5.84	No
	N4	109.23 ± 18.74	33.00 ± 5.29	No
